# Early Effects of Hyperbaric Oxygen on Inducible Nitric Oxide Synthase Activity/Expression in Lymphocytes of Type 1 Diabetes Patients: A Prospective Pilot Study

**DOI:** 10.1155/2019/2328505

**Published:** 2019-01-10

**Authors:** Ivana Resanovic, Zoran Gluvic, Bozidarka Zaric, Emina Sudar-Milovanovic, Aleksandra Jovanovic, Davorka Milacic, Radmilo Isakovic, Esma R. Isenovic

**Affiliations:** ^1^Institute of Nuclear Sciences Vinca, University of Belgrade, Laboratory of Radiobiology and Molecular Genetics, Belgrade, Serbia; ^2^Clinic for Internal Medicine, Zemun Clinical Hospital, School of Medicine, University of Belgrade, Belgrade, Serbia; ^3^Department of Hyperbaric Medicine, Zemun Clinical Hospital, Belgrade, Serbia

## Abstract

This study aimed at examining the early effects of hyperbaric oxygen therapy (HBOT) on inducible nitric oxide synthase (iNOS) activity/expression in lymphocytes of type 1 diabetes mellitus (T1DM) patients. A group of 19 patients (mean age: 63 ± 2.1) with T1DM and with the peripheral arterial disease were included in this study. Patients were exposed to 10 sessions of HBOT in the duration of 1 h to 100% oxygen inhalation at 2.4 ATA. Blood samples were collected for the plasma C-reactive protein (CRP), plasma free fatty acid (FFA), serum nitrite/nitrate, and serum arginase activity measurements. Expression of iNOS and phosphorylation of p65 subunit of nuclear factor-*κ*B (NF*κ*B-p65), extracellular-regulated kinases 1/2 (ERK1/2), and protein kinase B (Akt) were examined in lymphocyte lysates by Western blot. After exposure to HBOT, plasma CRP and FFA were significantly decreased (*p* < 0.001). Protein expression of iNOS and serum nitrite/nitrate levels were decreased (*p* < 0.01), while serum arginase activity was increased (*p* < 0.05) versus before exposure to HBOT. Increased phosphorylation of NF*κ*B-p65 at Ser^536^ (*p* < 0.05) and decreased level of NF*κ*B-p65 protein (*p* < 0.001) in lymphocytes of T1DM patients were observed after HBOT. Decreased phosphorylation of ERK1/2 (*p* < 0.05) and Akt (*p* < 0.05) was detected after HBOT. Our results indicate that exposure to HBO decreased iNOS activity/expression via decreasing phosphorylation of ERK1/2 and Akt followed by decreased activity of NF*κ*B.

## 1. Introduction

Diabetes mellitus (DM) type 1 (T1DM) is a multifactorial autoimmune disease associated with significant morbidity and mortality related to microvascular and macrovascular complications [1, 2]. T1DM is associated with abnormal synthesis of nitric oxide (NO) via activation of inducible NO synthase (iNOS) [3, 4], and an increased level of iNOS enzyme is associated with DM-related vascular complications [5–8]. The iNOS gene expression is stimulated through activation of transcription factors, such as nuclear factor-*κ*B (NF*κ*B). Furthermore, induction of iNOS is mediated via stimulation of NF*κ*B, by different stimuli including extracellular-regulated kinases (ERK1/2) and protein kinase B (Akt) [9, 10].

Systemic hyperbaric oxygen (HBO) therapy (HBOT) has been proposed as a medical treatment for DM patients with the developed peripheral arterial disease. HBOT is defined as therapeutic inhalation of 100% oxygen in the elevated pressure controlled conditions, which induces micro- and macrovascular hemodynamic changes [11]. An increase in oxygen arterial partial pressure in hyperbaric conditions promotes better solubility of plasma oxygen. This further results in the preservation of vitality of tissues, reversibly damaged by atherosclerosis-induced ischemia, simultaneously with microcirculation restoration [12]. Exposure to HBO decreases iNOS activity/expression followed by reduction of NO generation, which implies that this may be mechanisms responsible for the anti-inflammatory effect of HBOT [13, 14]. However, the exact molecular mechanism by which HBOT reduces inflammation remains still unclear.

We hypothesized that HBO affects iNOS regulation through inhibition of the NF*κ*B activation, by a mechanism that involves phosphorylation of ERK1/2 and Akt. The aim of this prospective pilot study was twofold: (1) to investigate the early effects of HBOT on iNOS activity/expression and (2) to investigate the involvement of Akt and ERK1/2 in the regulation of iNOS activity/expression, in T1DM patients exposed to HBO.

## 2. Material and Methods

### 2.1. Chemicals and Reagents

Lymphocyte separation media (LSM), Lymphosep, was obtained from BioWest S.A.S (Nuaillé, France). Protease inhibitor (Complete, Ultra, Mini, EDTA-free) and phosphatase inhibitor (PhosStop) cocktails were obtained from Roche (Mannheim, Germany). Luminol and p-coumaric acid were obtained from Sigma-Aldrich Corporation (St. Louis, MO, USA). The commercially available kit for determination of total cholesterol Beckman enzymatic reagent kit was purchased from Beckman Coulter (Brea, CA, USA). The nitrate/nitrite colourimetric assay kit was purchased from Cayman Chemical (Ann Arbor, MI, USA). For measuring the activity of arginase, we used L-arginine monohydrochloride obtained from Kemika (Zagreb, Croatia) and alpha-isonitrosopropiophenone (ISPF) obtained from Sigma-Aldrich Corporation (St. Louis, MO, USA). The polyclonal rabbit anti-iNOS and anti-NF*κ*B-p65, monoclonal mouse anti-actin antibody, secondary anti-mouse, and anti-rabbit IgG horseradish peroxidase-linked antibodies were obtained from Santa Cruz Biotechnology Inc. (Santa Cruz, CA, USA). The following antibodies were obtained from Cell Signaling Technology Inc. (Danvers, MA, US): anti-phospho-NF*κ*B-p65 (Ser^536^), anti-phospho-ERK1/2 (Thr^202^/Tyr^204^), anti-total ERK1/2, anti-phospho-Akt (Ser^473^), and anti-total Akt antibodies. The enhanced chemiluminescent reagent was obtained from Amersham, GE Healthcare (Buckinghamshire, UK).

### 2.2. Subjects and Methods

After obtaining the Ethical Committee of Zemun Clinical Hospital approval and written informed consent, 19 type 1 DM (T1DM) patients with no obvious DM ulcers, inflammatory redness on lower extremities, and contraindications for HBOT were included in this prospective pilot study during 2017, in Zemun Clinical Hospital, Zemun, Serbia. The study inclusion criterion was the presence of T1DM with the peripheral arterial disease. A total of 10 sessions of HBOT were applied as one session per day, for two weeks, in the duration of 1 h of 100% oxygen inhalation at 2.4 ATA. The demographic properties and medical history of all patients were recorded on the first visit, including gender, type of diabetes, current diabetes condition, hypertension, and other concomitant therapy of study interest (aspirin, statins, fibrates, antiplatelet therapy, angiotensin-converting enzyme inhibitors, angiotensin receptor blockers, calcium-channel blockers, and beta blockers). The peripheral arterial disease was confirmed by the standard questionnaire. Blood samples were collected, and lymphocytes were isolated and stored at −20°C. Half of the collected blood was transferred to EDTA-containing vacutainer tubes, incubated on ice for one hour, and subsequently centrifuged for 15 min at 2000 ×*g*. Obtained supernatants (plasma) were stored at −20°C before analysis. Serums were isolated by incubation of blood at room temperature for 30 min without anticoagulants, followed by 15 min centrifugation at 1800 ×*g*. Serums were stored at −20°C until further biochemical analysis. Glycosylated hemoglobin A1c (HbA_1c_), C-reactive protein (CRP), free fatty acids (FFA), nitrite/nitrate levels, and arginase activity were measured before and after completion of HBOT. Complete blood count, total cholesterol, and triglyceride (TG) were measured before HBOT.

### 2.3. Measurement of Serum HbA_1c_ Levels

The measurement of HbA_1c_ was carried out using an immunological method with an antibody to the beta chain of HbA_1c_ on Roche cobas c 501 analyzer (Roche Diagnostics, Indianapolis, IN, USA). The HbA_1c_ results are expressed as a percentage of glycosylated hemoglobin (%).

### 2.4. Measurement of Plasma TG and Total Cholesterol Concentration

The concentration of TG was measured using a commercially available kit according to the manufacturer's guidelines using a Roche cobas c 501 analyzer (Roche Diagnostics, Indianapolis, IN, USA). The concentration of total cholesterol was measured by standardized enzymatic colour test using Beckman Coulter Olympus AU400 analyzer (Brea, CA, USA) [15]. Total cholesterol and TG concentrations were expressed as mmol/L.

### 2.5. Measurement of Plasma CRP and FFA Concentrations

The concentration of CRP in the plasma was measured by the immunoturbidimetric method using a commercially available kit (system reagent for the quantitative determination of CRP in human plasma), following manufacturer guidelines, on Roche cobas c 501 analyzer (Roche Diagnostics, Indianapolis, IN, USA). CRP concentrations were expressed in mg/L and presented as % of CRP before HBOT.

The FFA concentration was measured in the plasma containing EDTA, using a modified version of the Duncombe method [16]. The principle of the method is that extracted FFA in chloroform, in the presence of an appropriate reagent (aqueous solution of Cu(NO_3_)_2_ × 3H_2_O with triethanolamine, pH 7.8), forms salts of copper, which in contact with diethyldithiocarbamate builds a yellow complex compound with a maximum absorbance at 436 nm [15]. The concentrations of FFA were expressed in mmol/L and presented as % of FFA before HBOT.

### 2.6. Measurement of Serum Nitrite/Nitrate Concentration and Arginase Activity

The concentration of the serum nitrite and nitrate was measured by using a nitrate/nitrite colourimetric assay kit (Cayman Chemical, Ann Arbor, MI, USA) according to the manufacturer's protocol [15]. The concentrations of nitrite/nitrate were expressed in mmol/L and presented as % of nitrite/nitrate before HBOT.

Arginase activity was measured spectrophotometrically in serum, according to the method of Corraliza et al. [17]. Arginase is the enzyme that converts L-arginine to urea and L-ornithine; thereby, the principle of this method is based on measurement of the amount of generated urea which is directly proportional to arginase activity. Briefly, aliquots of serum were incubated for 10 min at 55°C in complete assay mixture lacking arginine. The reaction was initiated by addition of L-arginine, and incubation was continued at 37°C for 1 h. The reaction was terminated by heating at 100°C for 45 min. Values of arginase activity were expressed in nmol/min/mg of proteins and presented as % of arginase activity before HBOT.

### 2.7. Isolation of Lymphocytes from Peripheral Blood

The heparinized whole blood samples from each patient were used for the isolation of lymphocytes with LSM, according to the method of Boyum [18]. The lymphocytes forming a layer directly above the LSM were isolated and washed twice with phosphate-buffered saline. Each wash was followed by centrifugation at 1200 ×*g* for 10 min. Finally, the supernatant was removed and stored at −70°C for further analysis.

### 2.8. Isolation of Proteins from Lymphocytes

Whole cell extracts were prepared by suspending cell pellets in ice-cold buffer for protein isolation (150 mM sodium chloride (NaCl), 20 mM tris(hydroxymethyl)aminomethane (TRIS), 2 mM ethylenediaminetetraacetic acid (EDTA), 2 mM dithiothreitol (DTT), 1% nonionic detergent Triton X-100, and 10% glycerol) containing phosphatase and protease inhibitor cocktails. Lymphocytes were lysed by rotation at +4°C for a period of 1 h. Lysed samples were then centrifuged for 30 min at 14000 ×*g* at +4°C. Protein concentration was determined by the Lowry method [19]. The supernatants were stored at −70°C for further analysis.

### 2.9. SDS-PAGE and Western Blotting

The lysate proteins (40 *μ*g/lane) were separated by 10% or 12% SDS-PAGE and transferred to PVDF membranes as previously described [20]. Membranes were blocked with 5% bovine serum albumin and probed with the following antibodies: anti-iNOS, NF*κ*B-p65 (Ser^536^), anti-NF*κ*B-p65, anti-phospho-Akt (Ser^473^), anti-total Akt, anti-phospho-ERK1/2 (Thr^202^/Tyr^204^), and anti-total ERK1/2. The membranes were washed and incubated with the appropriate secondary HRP-conjugated antibody and after that used for detection with the enhanced chemiluminescent reagent. Anti-actin monoclonal antibodies were used as a loading control. The obtained signals were quantified using ImageJ software (NIH, USA).

### 2.10. Statistical Analysis

Values are expressed as means ± SEM. SPSS 18.0 for Windows (SPSS Inc., Chicago, IL, USA) was used for all statistical calculations. Analysis of data was evaluated using two-tailed Student's *t*-test. The statistical significance level was set at *p* < 0.05.

## 3. Results

The anthropometric parameters including the metabolic parameters of T1DM patients at admission are presented in Table 1.

**Figure 1:**
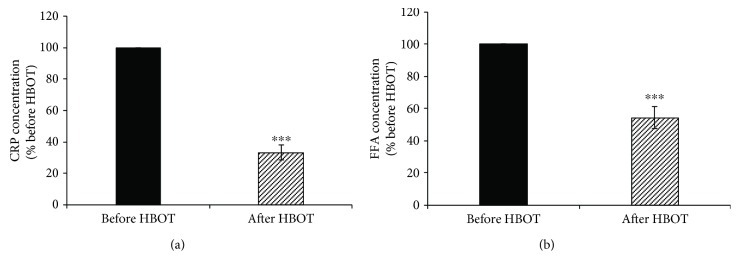
Effects of HBOT on plasma CRP and FFA levels. (a) The plasma level of CRP; (b) the plasma level of FFA. Results are expressed as % of the value obtained before HBOT and represent mean ± SEM (*n* = 15); ^∗∗∗^*p* < 0.001. CRP: C-reactive protein; FFA: free fatty acid. Other abbreviations are under Table 1.

Results presented on Figure 1(a) show a significant decrease (by 67%; *p* < 0.001) of plasma CRP concentration and plasma FFA concentration (by 46%; *p* < 0.001) after exposure to HBOT (Figure 1(b)).

**Figure 2:**
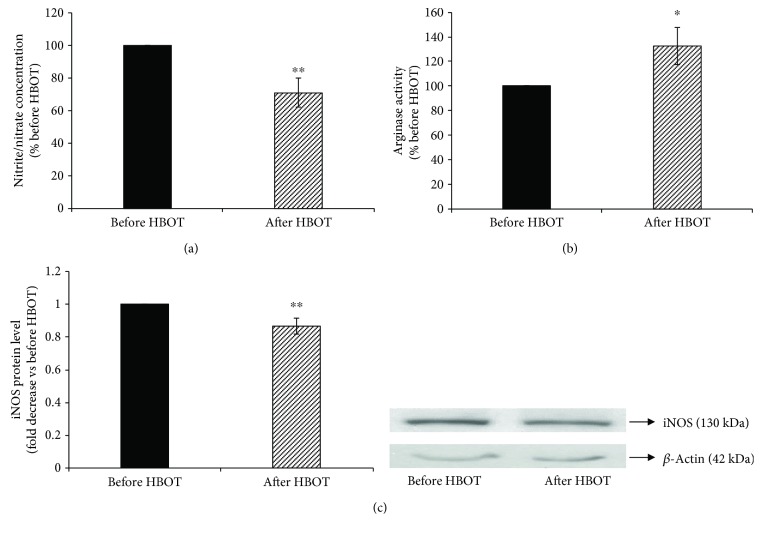
Changes in the serum nitrite/nitrate concentration, arginase activity, and iNOS protein level in lymphocytes after HBO exposure. (a) The serum nitrite/nitrate concentration was expressed as a % before HBOT; (b) the serum arginase activity was expressed as a % before HBOT; (c) level of iNOS protein in lymphocytes, normalized to *β*-actin and expressed as fold change before HBOT (arbitrary control set at 1). Inset: representative Western blots (*n* = 11–13); ^∗∗^*p* < 0.01; ^∗^*p* < 0.05. iNOS: inducible nitric oxide synthase. Other abbreviations are under Table 1.

The serum nitrite/nitrate concentration (Figure 2(a)) was decreased by 29% (before HBOT = 100%; after HBOT = 71 ± 8.9%; *p* < 0.01), while serum arginase activity was increased by 32% after exposure to HBOT (before HBOT = 100%; after HBOT = 132 ± 15.1%; *p* < 0.05) (Figure 2(b)). To assess a decrease in iNOS synthesis, protein samples from cell lysates were analyzed by Western blotting (Figure 2(c)). HBOT significantly decreased the iNOS protein level (before HBOT = 1-fold; after HBOT = 0.87 ± 0.05-fold, *p* < 0.01).

**Figure 3:**
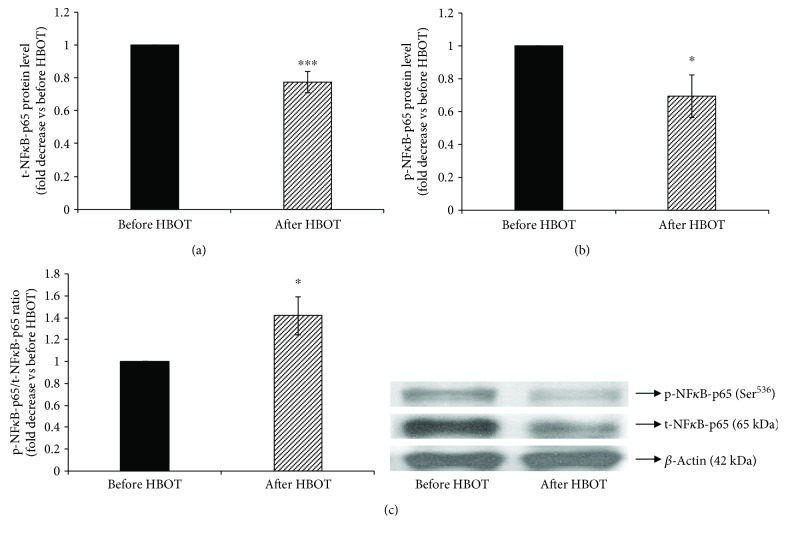
Phosphorylation of NF*κ*B-p65 at Ser^536^ in lymphocytes after HBO exposure. (a) Level of t-NF*κ*B-p65 protein in lymphocytes, normalized to *β*-actin and expressed as fold change before HBOT (arbitrary control set at 1); (b) level of p-NF*κ*B-p65 (Ser^536^) protein in lymphocytes, normalized to *β*-actin and expressed as fold change before HBOT (arbitrary control set at 1); (c) ratio of p-NF*κ*B-p65/t-NF*κ*B-p65 expressed as fold change before HBOT (arbitrary control set at 1). Inset: representative Western blots (*n* = 5–15); ^∗∗^*p* < 0.01; ^∗^*p* < 0.05; p-NF*κ*B-p65: the phosphorylated form of p65 subunit of nuclear factor-*κ*B; t-NF*κ*B-p65: the total form p65 subunit of nuclear factor-*κ*B. Other abbreviations are under Table 1.

Since the promoter of iNOS gene is the binding site for NF*κ*B transcription factor [21], we further examined whether NF*κ*B is involved in HBOT downregulation of iNOS activity/expression by measuring p65 subunit level of NF*κ*B (Figure 3). Furthermore, we also assessed the effect of HBOT of the phosphorylation state of NF*κ*B-p65 at Ser^536^ by the immunoblotting with the phospho-specific NF*κ*B-p65 antibody that recognizes NF*κ*B-p65 only when phosphorylated at Ser^536^.

HBOT leads to a significant decrease of the level of the total form of NF*κ*B-p65 by 0.23-fold (before HBOT = 1-fold; after HBOT = 0.77 ± 0.07-fold, *p* < 0.01) (Figure 3(a)), as well as the level of the phosphorylated form of NF*κ*B-p65 by 0.3-fold (before HBOT = 1-fold; after HBOT = 0.70 ± 0.13-fold, *p* < 0.05) (Figure 3(b)). However, HBOT increased the ratio between the phosphorylated and total forms of NF*κ*B-p65 in the lysate of T1DM patients' lymphocytes (before HBOT = 1-fold; after HBOT = 1.42 ± 0.17-fold, *p* < 0.05) (Figure 3(c)).

**Figure 4:**
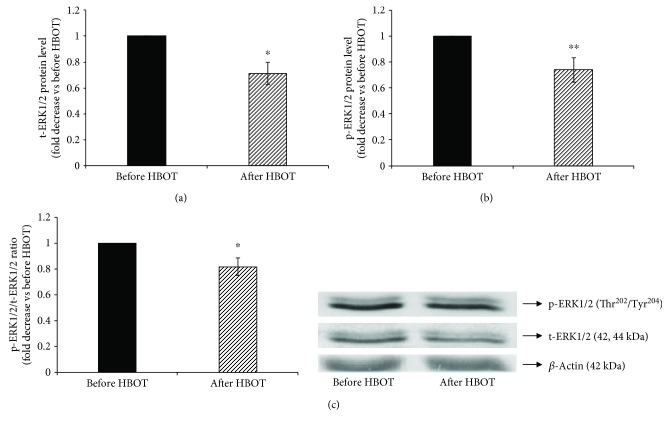
Phosphorylation of ERK1/2 at Thr^202^ and Tyr^204^ in lymphocytes after HBO exposure. (a) Level of t-ERK1/2 protein in lymphocytes, normalized to *β*-actin and expressed as fold change before HBOT (arbitrary control set at 1); (b) level of p-ERK1/2/(Thr^202^/Tyr^204^) protein in lymphocytes, normalized to *β*-actin and expressed as fold change before HBOT (arbitrary control set at 1); (c) ratio of p-ERK1/2/t-ERK1/2 expressed as fold change before HBOT (arbitrary control set at 1). Inset: representative Western blots (*n* = 14); ^∗∗^*p* < 0.01; ^∗^*p* < 0.05; p-ERK1/2: the phosphorylated form of extracellular signal-regulated kinases 1 and 2; t-ERK1/2: the total form of extracellular signal-regulated kinases 1 and 2. Other abbreviations are under Table 1.

**Figure 5:**
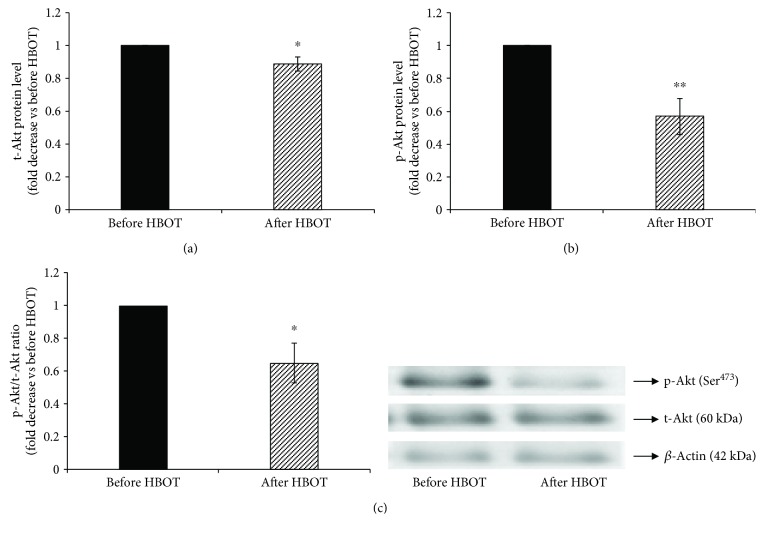
Phosphorylation of Akt at Ser^473^ in lymphocytes after HBO exposure. (a) Level of t-Akt protein in lymphocytes, normalized to *β*-actin and expressed as fold change before HBOT (arbitrary control set at 1); (b) level of p-Akt (Ser^473^) protein in lymphocytes, expressed as fold change before HBOT (arbitrary control set at 1); (c) ratio of p-Akt/t-Akt expressed as fold change before HBOT (arbitrary control set at 1). Inset: representative Western blots (*n* = 7); ^∗∗^*p* < 0.01; ^∗^*p* < 0.05; p-Akt: the phosphorylated form of protein kinase B; t-Akt: the total form of protein kinase B. Other abbreviations are under Table 1.

Since we have previously shown that the stimulation of iNOS also involves activation of ERK1/2 phosphorylation [22] and activation of Akt phosphorylation [23], we next examined whether ERK1/2 and Akt are involved in decreased iNOS activity/expression after the exposure to HBOT. Therefore, we assessed the effect of HBOT of the phosphorylation state of ERK1/2 (Figure 4) and Akt (Figure 5) by the immunoblotting with phospho-specific ERK1/2 and Akt antibodies that recognize ERK1/2 and Akt only when ERK is phosphorylated at Thr^202^/Tyr^204^ and Akt at Ser^473^, respectively.

HBOT decreased the total ERK1/2 protein level (before HBOT = 1-fold; after HBOT = 0.89 ± 0.05-fold; *p* < 0.05) (Figure 4(a)) and total Akt protein level, normalized to *β*-actin (before HBOT = 1-fold; after HBOT = 0.89 ± 0.04-fold; *p* < 0.05) (Figure 5(a)). Similarly, HBOT decreased phosphorylation of ERK1/2 (Figures 4(b) and 4(c)) (before HBOT = 1-fold; after HBOT = 0.79 ± 0.06-fold; *p* < 0.01) and phosphorylation of Akt (Figures 5(b) and 5(c)) (before HBOT = 1-fold; after HBOT = 0.57 ± 0.11-fold; *p* < 0.01). These results suggest that exposure to HBOT significantly decreases iNOS activity/expression and indicate that ERK1/2 and Akt are involved in HBOT-downregulated iNOS activity/expression in T1DM patients.

## 4. Discussion

The principal findings of our study included the observation that in T1DM patients, HBOT downregulates the iNOS activity/expression by decreasing phosphorylation of ERK1/2 and Akt followed by a decreased activation of NF*κ*B. For this study, we have used HBOT as a medical treatment for T1DM patients with the developed peripheral arterial disease, with the purpose to reduce inflammation and to promote microcirculation.

Biochemical profile of DM is commonly presented by elevated levels of HbA_1c_, CRP, FFA, and NO which provide an environment of oxidative stress and inflammation [24]. HbA_1c_ is a reliable biomarker for the diagnosis and prognosis of DM and correlates well with the risk of long-term diabetes complications [25]. Results from our study show that the level of HbA_1c_ was 8.6 ± 0.08%, which indicates a higher risk of DM-associated complications [26].

Elevated FFA, which is common in the diabetic population [27], may trigger systemic inflammation and impair NO bioavailability, leading to impaired endothelium-dependent and insulin-mediated vasodilation [28]. Increase in FFA level is associated with an increase in NO level and upregulation of iNOS mRNA as well as the reduction of insulin output in cultured prediabetic rat pancreatic islets [29]. Also, increased plasma FFA levels are accompanied by increased expression of several NF*κ*B-dependent cytokines, leading to chronic inflammatory processes [30]. In our study, we observed a significant decrease in FFA after HBOT in T1DM patients. These results are consistent with data gained by Teshigawara et al. [31]. Teshigawara et al. showed that FFA concentration was significantly decreased after HBOT, implying that HBOT could have beneficial effects on lipid metabolism. Clinical manifestation of inflammation is characterized by the increase of acute-phase proteins, among which prominent place has CRP [32, 33]. The level of CRP is a well-known marker of inflammation [32], and it is reported that CRP may stimulate iNOS expression and NO production [34]. Our results show that exposure to HBOT significantly reduces the level of CRP in T1DM patients. Thus, the results from our study suggest that HBOT leads to a reduction of inflammation and subsequent progression of atherosclerosis [35]. iNOS is an important mediator of inflammation and may be the critical link between metabolic disorders and inflammation [36].

iNOS expression occurs after induction as a response to cytokines and other proinflammatory agents. After induction, iNOS continuously produces 100- to 1000-fold more NO than constitutive NOS [37, 38]. Excessive NO production favors the formation of peroxynitrite and thus contributes to tissue injury and reduced NO bioavailability [39, 40]. In our study, we have observed that HBOT leads to a decrease in iNOS expression in lymphocytes and consequently decreases the level of the serum nitrite/nitrate concentration. Our results also show that HBOT increased serum arginase activity, and increased arginase activity may reduce the substrate availability of NOS, since NOS and arginase use the same substrate, the amino acid L-arginine, and consequently could reduce nitrite/nitrate concentration [41].

Moreover, numerous literature data reported that activities of iNOS and arginase are regulated reciprocally by cytokines and that the inhibition of iNOS leads to the subsequent decrease in the NO production that is associated with increased arginase activity [42–44]. Elevated levels of glucose in diabetes may enhance NO production through increased expression of endothelial NOS (eNOS) and iNOS gene and protein levels [8, 45–47]. Although iNOS/eNOS might be responsible for the release of NO from endothelial cells [47], numerous literature data reported that hyperglycaemia increased the NO level mostly through activation of iNOS [6, 48–50]. Thus, the decreased serum nitrite/nitrate concentration suggests that decreased iNOS and increased arginase activity after exposure to HBOT in our study may be due to decreased iNOS expression. This is in accordance with other results [51–56]. Pedoto et al. have also observed in their study that HBO pretreatment reduces lipopolysaccharide-induced iNOS expression [51]. Also, HBOT reduces the level of exhaled NO for 1 h in human subjects [55]. Similarly, rats exposed to HBOT for 7 days show significantly decreased NOS activity [13], while rabbits exposed to HBOT for 5 days have decreased expression of iNOS [52].

The results from our study reveal that HBOT significantly increased phosphorylation of NF*κ*B-p65 at Ser^536^, while it decreased the level of NF*κ*B-p65 protein in lymphocytes of T1DM patients. Our results are in agreement with the results of Meng et al. who have demonstrated that exposure of rats to HBO significantly reduces the activation of the NF*κ*B protein [57]. Mattioli et al. reported that phosphorylation of NF*κ*B-p65 at Ser^536^ negatively regulates the import of NF*κ*B-p65 to the nucleus [58]. Additionally, phosphorylation of NF*κ*B-p65 at Ser^536^ plays an important role in promoting the proteasomal degradation of NF*κ*B-p65, thus leading to transcriptional termination of NF*κ*B-dependent genes [59], and phosphorylation of NF*κ*B-p65 at Ser^536^ regulates the ability of nuclear I*κ*B*α* to inhibit the NF*κ*B binding to promoters in human leukocytes and thereby inhibit transcription of genes in a selective manner [60]. Furthermore, phosphorylation of NF*κ*B-p65 at Ser^536^ increases turnover of NF*κ*B-p65, thereby inhibiting NF*κ*B activity in macrophages and subsequently reducing inflammation [61]. Since iNOS expression is regulated primarily by control of gene transcription, a possible explanation for the decrease of iNOS protein level after HBOT may be that decreased activation of transcription factor NF*κ*B-p65 subunit, which has a binding site on the iNOS gene promoter, consequentially leads to reduced iNOS expression. Here, we demonstrate that HBOT causes downregulation of NF*κ*B-p65 protein and this decrease could be because of the decreased cytokine level after exposure to HBO [62]. Thus, we assume that HBOT downregulates iNOS activity/expression by a mechanism involving NF*κ*B in combination with decreased CRP concentrations. Although CRP by itself did not affect NO synthesis, CRP further enhanced cytokine-evoked increases in iNOS mRNA protein levels [34]. Also, we postulate that decreased iNOS expression is associated with decreased expression of FFA translocase, a cluster of differentiation 36 (CD36), and a reduction in the level of FFA after exposure to HBOT.

ERK1/2 is involved in the regulation of iNOS activation [63–66]. Increased ERK1/2 phosphorylation upregulates both NF*κ*B activity and iNOS protein expression [64, 67–70], while inhibition of ERK1/2 phosphorylation downregulates the expression of cytokine-induced NF*κ*B-activation [71]. Furthermore, inhibition of ERK1/2 decreases iNOS gene expression [71]. In our study, HBOT significantly decreases the phosphorylation of ERK 1/2, and this decrease could be due to the decreased NF*κ*B activity [9]. Our results are in accordance with the study of Niu et al. [72]. Niu et al. reported that HBO suppressed the phosphorylation of ERK1/2 and decreased the synthesis of NO. Therefore, we assume that decreases in ERK1/2 phosphorylation after exposure to HBOT could lead to the reduction of NF*κ*B activation and consequentially reduced expression of iNOS protein.

Akt is an upstream activator of NF*κ*B, whereby phosphorylated Akt (or ERK1/2) activates NF*κ*B inhibitor kinase and consequently leads to transcription and translation of iNOS [68, 73]. Hattori et al. reported that the activation of NF*κ*B is suppressed by inhibitors of Akt [74]. In our study, HBOT decreased Akt phosphorylation, indicating that activation of Akt is involved in mediating the effect of HBOT on the regulation of iNOS. Our results are in accordance with the study of Felderhoff-Mueser et al. who reported that hyperoxia reduces phosphorylation of Akt at Ser^473^ [75]. Since the phosphorylation of Akt is necessary for NF*κ*B activation, we suggest that a decrease in Akt phosphorylation leads to NF*κ*B downregulation and a decrease in iNOS protein expression.

**Figure 6:**
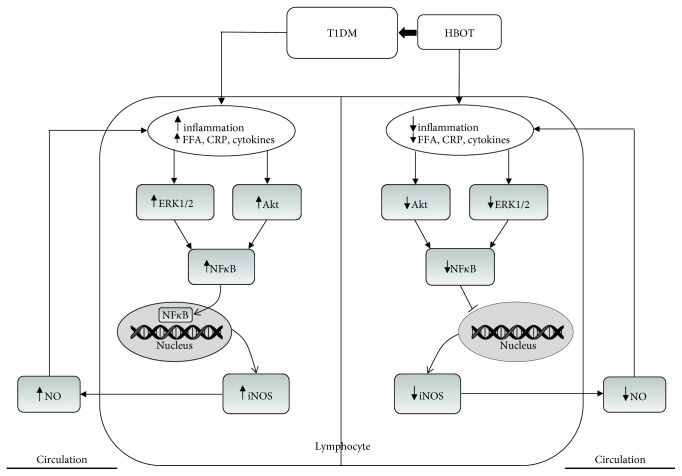
The proposed mechanism by which HBOT affects regulation of iNOS activity/expression and nitrite/nitrate production in T1DM patients. iNOS: inducible nitric oxide synthase; NF*κ*B-p65: the p65 subunit of nuclear factor-*κ*B; NO: nitric oxide; ERK1/2: extracellular signal-regulated kinases 1 and 2; Akt: protein kinase B; CRP: C-reactive protein; FFA: free fatty acid. Other abbreviations are under Table 1.

To explain our results, we proposed the following model (Figure 6). Altered level of FFA, CRP, and cytokines caused by T1DM induces inflammation which influences the iNOS/NO pathway and thus promotes vascular complications by attenuating insulin action. This is likely a consequence of increased ERK1/2/Akt activation, which further upregulates NF*κ*B. There are several possibilities to explain the effect of HBOT in the modulation of iNOS. It is possible that HBOT leads to the decreased level of CRP, FFA, and cytokines, and consequently this leads to decreased activation of ERK1/2 and Akt activation and thus decreases the activity of NF*κ*B, and subsequently iNOS or NF*κ*B activation by cytokines in T1DM is achieved only through Akt. Also, NF*κ*B-p65 activation could be involved in ERK1/2 activation [9].

In accordance to the promising results of this study that pointed out the early effects of HBOT on some molecular aspects of inflammation in T1DM patients, we are planning to carry out another study that will be performed on a more significant sample size with inclusion of patients suffering from metabolic syndrome and type 2 DM as well healthy control subjects. The currently presented prospective pilot study is based on the number of administered HBOT sessions included in one HBOT course and the exploration of some early inflammatory molecular effects of HBOT after one course of exposure. As the positive effects are detected in our study, the evaluation of long-term HBOT effects (e.g., after 1 or 5 years) regarding the molecular basis of the anti-inflammatory effects of the therapy is needed. Furthermore, there is a possibility to test the effects of different HBOT administration protocols and to select the most appropriate model (i.e., study plan could be performed either through the increase in the number of sessions involved in HBOT course or by shortening of the period between successive HBOT courses). Besides evaluating the molecular mechanisms involved in the reduction of inflammation, it is also necessary to design prediction models for major cardio- and cerebrovascular atherosclerotic events and to examine the influence of various confounding variables that will be of great interest and contribution in the planning of primary and secondary prevention of major atherosclerotic accidents in diabetic patients and in improving patients' quality of life.

The limitation of our study is a small number of T1DM patients exposed to HBOT. The other limitation of our study is that we did not directly measure the level of cytokines and we did not measure the level of NO in lymphocytes and the level of NF*κ*B in the lymphocyte nucleus.

In conclusion, in the present study, we demonstrate for the first time that HBOT causes downregulation of iNOS activity/expression by a mechanism involving ERK1/2, Akt, and NF*κ*B, in T1DM patients. The results from our study suggest that HBOT has a vital role in the reduction of inflammation in T1DM patients. Future studies on a larger population size are needed to elucidate the molecular mechanism by which HBOT regulates iNOS activity/expression in T1DM patients with vascular complications.

## Figures and Tables

**Table 1:** Anthropometric and metabolic parameters of T1DM patients. Results are presented as mean ± SEM*:* HBOT: hyperbaric oxygen therapy; T1DM: type 1 diabetes mellitus; BMI: body mass index; HbA1c: glycosylated hemoglobin; WBC: white blood cells; RBC: red blood cells; TG: triglyceride.

Parameter	Mean ± SE (min–max)	Reference range
Age (years)	63 ± 2.1 (50–80)	
Weight (kg)	82.6 ± 2.9 (62–99)	
High (m)	1.74 ± 0.02 (1.6–1.8)	
BMI (kg/m)	27.4 ± 0.9 (21.8–33.7)	
HbA1c (%)	8.6 ± 0.08 (6.1–12.7)	4.8–5.9
Hemoglobin (g/L)	136 ± 3.4 (111–169)	120–175
Hematocrit (%)	40 ± 0.1 (30–50)	35–50
WBC (×10^3^/L)	8.3 ± 0.5 (3.4–11.3)	3.5–10.5
RBC (×10^6^/L)	4.7 ± 0.2 (3.9–6.6)	3.9–5.7
Platelets (×10^3^/L)	264 ± 26.7 (127–393)	150–450
Cholesterol (mmol/L)	5 ± 0.4 (3.25–7.9)	5-6.2
TG (mmol/L)	2.3 ± 0.4 (0.7–4.0)	1.7–2.2

## Data Availability

The data used to support the findings of this study are available from the corresponding author upon request.

## Abbreviations

Akt: Protein kinase B
CD36: Cluster of differentiation 36
CRP: C-reactive protein
DM: Diabetes mellitus
DTT: Dithiothreitol
EDTA: Ethylenediaminetetraacetic acid
eNOS: Endothelial NOS
ERK1/2: Extracellular signal-regulated kinases 1/2
FFA: Free fatty acids
HbA_1c_: Glycosylated hemoglobin A1c
HBO: Hyperbaric oxygen
HBOT: Hyperbaric oxygen therapy
iNOS: Inducible nitric oxide synthase
ISPF: Alpha-isonitrosopropiophenone
LSM: Lymphocyte separation media
NaCl: Sodium chloride
NF*κ*B-p65: p65 subunit of nuclear factor-*κ*B
NO: Nitric oxide
T1DM: Type 1 diabetes mellitus
TES: N-[Tris(hydroxymethyl)methyl]-2-aminoethanesulfonic acid
TG: Triglyceride
TRIS: Tris(hydroxymethyl)aminomethane.
